# Exosomal ANGPTL1 attenuates colorectal cancer liver metastasis by regulating Kupffer cell secretion pattern and impeding MMP9 induced vascular leakiness

**DOI:** 10.1186/s13046-020-01816-3

**Published:** 2021-01-07

**Authors:** Kai Jiang, Haiyan Chen, Yimin Fang, Liubo Chen, Chenhan Zhong, Tongtong Bu, Siqi Dai, Xiang Pan, Dongliang Fu, Yucheng Qian, Jingsun Wei, Kefeng Ding

**Affiliations:** 1 Department of Colorectal Surgery and Oncology, Key Laboratory of Cancer Prevention and Intervention, Ministry of Education, the Second Affiliated Hospital, Zhejiang University School of Medicine, Hangzhou, Zhejiang China; 2 Cancer Institute, Key Laboratory of Cancer Prevention and Intervention, Ministry of Education, The Second Affiliated Hospital, Zhejiang University School of Medicine, Hangzhou, Zhejiang China; 3 Department of Medical Oncology, Key Laboratory of Cancer Prevention and Intervention, Ministry of Education, The Second Affiliated Hospital, Zhejiang University School of Medicine, Hangzhou, Zhejiang China

**Keywords:** Exosomal ANGPTL1, Colorectal Cancer, Liver metastasis, PMNs, Kupffer cell, MMP9

## Abstract

**Background:**

Angiopoietin-like protein 1 (ANGPTL1) has been proved to suppress tumor metastasis in several cancers. However, its extracellular effects on the pre-metastatic niches (PMNs) are still unclear. ANGPTL1 has been identified in exosomes, while its function remains unknown. This study was designed to explore the role of exosomal ANGPTL1 on liver metastasis in colorectal cancer (CRC).

**Methods:**

Exosomes were isolated by ultracentrifugation. The ANGPTL1 level was detected in exosomes derived from human CRC tissues. The effects of exosomal ANGPTL1 on CRC liver metastasis were explored by the intrasplenic injection mouse model. The liver PMN was examined by vascular permeability assays. Exosomal ANGPTL1 localization was validated by exosome labeling. The regulatory mechanisms of exosomal ANGPTL1 on Kupffer cells were determined by RNA sequencing. qRT-PCR, Western Blot, and ELISA analysis were conducted to examine gene expressions at mRNA and protein levels.

**Results:**

ANGPTL1 protein level was significantly downregulated in the exosomes derived from CRC tumors compared with paired normal tissues. Besides, exosomal ANGPTL1 attenuated liver metastasis and impeded vascular leakiness in the liver PMN. Moreover, exosomal ANGPTL1 was mainly taken up by KCs and regulated the KCs secretion pattern, enormously decreasing the MMP9 expression, which finally prevented the liver vascular leakiness. In mechanism, exosomal ANGPTL1 downregulated MMP9 level in KCs by inhibiting the JAK2-STAT3 signaling pathway.

**Conclusions:**

Taken together, exosomal ANGPTL1 attenuated CRC liver metastasis and impeded vascular leakiness in the liver PMN by reprogramming the Kupffer cell and decreasing the MMP9 expression. This study suggests a suppression role of exosomal ANGPTL1 on CRC liver metastasis and expands the approach of ANGPTL1 functioning.

**Supplementary Information:**

The online version contains supplementary material available at 10.1186/s13046-020-01816-3.

## Background

Colorectal cancer (CRC) is one of the most common digestive tract malignancies worldwide with high prevalence and mortality [1]. Distant metastasis is the leading cause of cancer-associated death in CRC patients [2]. The 5-year survival rate is 92% for patients with local disease, while it sharply declines to 53 and 11% for patients with regional and distant metastasis [3]. The liver is the most common site of CRC metastasis. It is reported that ≤25% of CRC patients have synchronous colorectal liver metastases (CLM) upon diagnosis, and up to half of CRCs will eventually lead to liver metastasis [4]. Despite advances in surgical technique and targeted therapy, CLM patients’ prognosis is still poor [5]. It is urgent to explore the mechanism of liver metastasis and seek a new strategy for CLM treatment.

In our previous study [6], we found some of the angiopoietin-like proteins (ANGPTLs) were downregulated in CRC tissue, among which ANGPTL1 was the most significant one. ANGPTLs are a family of proteins similar to angiopoietins in structure, including ANGPTL1 to ANGPTL8 [7]. They were reported to affect angiogenesis [7], inflammation [8], metabolism disorders [9], hematopoiesis [10], and cancer development [11, 12]. Early studies showed that Angiopoietin-like protein 1 (ANGPTL1) acts as an antiangiogenic factor and a tumor suppressor [13, 14]. ANGPTL1 is downregulated in various cancers [6, 7], and several studies have proved its inhibitory role in tumor growth and metastasis [15–18]. Our previous study also demonstrated that ANGPTL1 overexpression inhibited the migration and invasion of CRC cells, leading to liver metastasis suppression. Besides, low expression of ANGPTL1 was related to poorer prognosis in CRC patients [6].

Nevertheless, previous researches about ANGPTL1’s function limited in primary tumors [6, 16, 18]. As a secretory protein [14], the biological effects of extracellular ANGPTL1 on the metastatic organs are still under investigation. Increasing evidence shows that the primary tumor-secreted factors and exosomes can enhance metastasis by promoting a supportive microenvironment in the metastatic organs, named the pre-metastatic niches (PMNs). The PMNs include several characteristics, such as vascular leakiness, inflammation, and immunosuppression [19]. Exosomes are small extracellular vesicles ranging from 50 to160 nm in size carrying proteins, nucleic acids, and lipids [20]. Recently, tumor-derived exosomes have been reported to be involved in PMNs formation [21, 22]. As the earliest event during PMNs evolution, vascular permeability is always regulated by tumor-derived exosomes [23]. For instance, CRC-derived exosomal miR-25-3p can promote PMNs formation by inducing vascular permeability [24]; breast cancer-derived exosomal miR-105 can destroy vascular endothelial barriers to promote metastasis [25]. Interestingly, ANGPTL1 has been identified in exosomes derived from saliva [26], urine [27], and ovarian cancer cells [28]. Nevertheless, the function of exosomal ANGPTL1 in CRC is still unknown.

In this study, we focused on the role of exosomal ANGPTL1 in CRC metastasis. We studied the protein level of ANGPTL1 in exosomes derived from tumor and normal tissues in CRC patients. Both In vivo and in vitro models were applied to characterize the effects of exosomal ANGPTL1 on CRC liver metastasis and PMNs formation. We demonstrated that exosomal ANGPTL1 attenuates CRC liver metastasis by regulating the kupffer cell secretion pattern and impeding vascular leakiness in the liver PMN.

## Methods

### Patients and specimens

The clinical CRC and paired normal tissues were obtained from CRC patients (*n* = 8) in the Second Affiliated Hospital of Zhejiang University School of Medicine. This project was approved by the ethical committee of the Second Affiliated Hospital of Zhejiang University School of Medicine. Informed consent was obtained from all patients.

### Cell culture

The human colorectal cancer cell line SW620 and Human umbilical vein endothelial cell (HUVEC) were obtained from the American Type Culture Collection (Rockville, MD, USA). The ANGPTL1 stably overexpressed cell line (SW620-ANGPTL1) and its control cell line (SW620-Ctrl) were established in the previous study [6]. The Immortalized Mouse Kupffer Cell line (ImKC) was purchased from MilliporeSigma (USA). SW620 cells and HUVECs were cultured in RMPI-1640 medium (Gibco, Carlsbad, CA) supplemented with 10% fetal bovine serum (FBS, Gibco, Brazil). ImKC were cultured in DMEM medium (Gibco, Carlsbad, CA) supplemented with 10% FBS (Gibco, Brazil).

### Exosome collection and characterization

Exosomes were collected by sequential ultracentrifugation. CRC cells were cultured in the exosome-depleted (160,000×g, 16 h) complete medium for 72 h. The supernatants were collected and centrifugate at 500×g for 10 min to remove cell contamination, then at 3000×g for 20 min to remove apoptotic bodies and large cell debris, followed by centrifugation at 12,000×g for 20 min to remove large microvesicles. Next, exosomes were collected by 100,000×g centrifugation for 70 min (Beckman Ti70). The exosome pellet was resuspended in 20 mL of phosphate-buffered saline (PBS) and collected by 100,000×g ultracentrifugation for 70 min (Beckman Ti70). Exosome preparation was verified by Transmission electron microscopy (TEM). Exosome size was measured by dynamic light scattering (DLS) analysis using Zetasizer Nano ZSE (Malvern Panalytical, Shanghai, China).

For tissue-derived exosome collection, the CRC tumors and paired normal tissues were cut into 1 mm × 1 mm pieces and cultured in 15 mL of FBS-free RMPI-1640 medium for 24 h. Then the supernatant was harvested for further isolation of exosomes.

### Exosome treatment and labeling

Purified exosomes were injected into the mouse retro-orbital venous sinus in a total volume of 100 μL PBS. For in vivo education experiments, mice received 5 μg of exosomes every other day for 21 days. Retro-orbital injection of PBS was used in control groups. For in vitro education, exosomes (10 μg/mL) were added into the culture medium (CM) of ImKC for 3, 6, 12, 24 h. For exosome-tracking experiments, exosomes were labeled using PKH67 membrane dye (Sigma, Shanghai, China), followed by 100,000×g ultracentrifugation for 70 min, and labeled exosomes were resuspended in PBS. In experiments involving the evaluation of exosome incorporation, labeled exosomes were injected retro-orbitally into the mice or added into the CM of ImKC 24 h before immunofluorescence analysis for exosome cells.

### Animal model

To analyze the role of exosomal ANGPTL1 in CRC liver metastasis, we pre-educated the 6–8-week-old scid-beige mice (SLAC Laboratory Animal Co. Ltd., Shanghai, China) retro-orbitally with exosomes for 21 days (5μg/100ul, every other day). Then, 2 × 10^6^ SW620 cells were injected into the mice spleen as described in the previous study [29, 30]. A small animal IVIS Lumina Imaging System (Caliper Life Sciences, Hopkinton, MA) was used for liver lesion monitor. All mice were sacrificed at one month, and the livers were harvested for Hematoxylin and eosin (H&E) staining and analysis of metastases. For liver macrophage elimination, liposome clodronate was injected via the tail vein in a dose of 0.2 mL/20-25 g as a tool to suppress macrophage function by inducing apoptosis [31]. Liposomes containing PBS were injected as a control. All animal experiments were approved by the Institutional Ethics Committee of the Second Affiliated Hospital Zhejiang University School of Medicine.

### In vivo vascular permeability assay

After pre-education with exosomes for 21 days, mice were injected with the recombinant mouse MMP9 (rmMMP9; 50 μg/kg body weight; R&D) intravenously. The rmMMP9 was preactivated using 1 mM aminophenylmercuricacetate (AMPA, Sigma, Shanghai, China) for 2 h at 37 °C. One hour after rmMMP9 injection, FITC-Dextran (~70KD; 100 mg/kg; Sigma, Shanghai, China) was injected through the tail vein. After one hour, mice were sacrificed and perfused with PBS to remove the excess dye. Liver tissues were embedded in Tissue-Tek O.C.T. Compound (Sakura; Torrance, CA, USA) to make frozen blocks for sectioning and immunofluorescent staining. Stained sections were viewed and photographed with a fluorescence microscope. The intensity of fluorescence was measured using ImageJ software (ImageJ software v1.8.0).

### Endothelial permeability

HUVECs (2 × 10^4^) were seeded on transwell filters (0.4 μm pore size; Corning, Shanghai, China). After reaching confluence, HUVECs were treated with CM from ImKC educated by PBS, Ctrl-Exo, or ANGPTL1-Exo (with or without rmMMP9, 100 ng/mL) for 48 h. Then, FITC-Dextran (1 mg/mL) was added to the top well. 40 μL medium in the bottom well was taken for fluorescence measurement every 30 min using a SpectraMax microplate reader (SpectraMax i3, Molecular Devices, USA) at 488 nm excitation and 520 nm emission. The fluorescence intensity represents the passage of FITC-Dextran, which represents the the HUVECs layer permeability.

### Small interfering (siRNA) transfection

Mouse MMP9 siRNA was purchased from Tsingke biological technology (Hangzhou, China). The MMP9 siRNA and scramble siRNA were added into the ImKC 24 h after exosome pre-education. The siRNA transfection was performed with Lipofectamine 3000 (Invitrogen, Carlsbad, CA) according to the manufacturer’s instructions. The siRNA sequences were as follows: MMP9 siRNA, 5′-CAAGACAAAGCCUAUUUCUTT-3′ (sense), 5′-AGAAAUAGGCUUUGUCUUGTT-3′ (antisense).

### qRT-PCR

Total RNAs were isolated from cells and mouse livers with TRIzol reagent (Invitrogen, USA) and evaluated using NanoDrop 2000 spectrophotometer (Thermo Scientific, Pittsburgh, PA, USA). qRT-PCR was conducted using a standard SYBR-Green PCR kit protocol (YEASEN, Shanghai, China) with a 7500 Fast Real-Time PCR System (Life Technologies, Shanghai, China). The primers were synthesized by Tsingke biological technology (Hangzhou, China). The sequences of all primers are listed in Table 1.

**Table 1:** Primers used for quantitative real-time PCR were (5′ – 3′)

Genes	Forward	Reverse
*Gapdh*	GGTGAAGGTCGGTGTGAACG	CTCGCTCCTGGAAGATGGTG
*Lif*	ATTGTGCCCTTACTGCTGCTG	GCCAGTTGATTCTTGATCTGGT
*Il-1a*	CGCTTGAGTCGGCAAAGAAAT	CTTCCCGTTGCTTGACGTTG
*Ccl5*	GCTGCTTTGCCTACCTCTCC	TCGAGTGACAAACACGACTGC
*Csf3*	GCACTATGGTCAGGACGAGAG	GGGGAAATACCCGATAGAGCC
*Mmp9*	TGTCTGGAGATTCGACTTGAAGTC	TGAGTTCCAGGGCACACCA
*Cxcl2*	CCAACCACCAGGCTACAGG	GCGTCACACTCAAGCTCTG
*ZO1*	TTCGTACCTGGCATTGACTGG	TTCGTACCTGGCATTGACTGG
*CLND5*	CTCTGCTGGTTCGCCAACAT	CAGCTCGTACTTCTGCGACA

### RNA sequencing

Total RNA of ImKC cells educated by PBS or exosomes for 24 h in vitro was extracted and subjected to BGI (Huada Genomics Institute Co. Ltd., Guangzhou, China) for RNA sequencing. The DEGseq R package was used to analyze differentially expressed genes based on the conditions of a fold change (FC) ≥ 1 and Q-values ≤0.001. Gene Ontology (GO) and Kyoto Encyclopedia of Genes and Genomes (KEGG) pathway enrichment analyses were performed to explore the significant pathways. All mRNA sequencing data were uploaded to NCBI (accession number PRJNA656088).

### Western blot and ELISA

The tissue lysates were prepared using RIPA lysis buffer, while the cell and exosome lysates were prepared using 3× Loading Buffer. All samples were performed as depicted previously [6]. Exosome proteins were detected with the antibodies as follows: Alix (1:1000, Cell Signaling Technology, Beverly, MA, USA), Calnexin (1:1000, Cell Signaling Technology, Beverly, MA, USA), CD9 (1:500, Santa Cruz Biotechnology, Dallas, Texas, USA), ANGPTL1 (1:500, Santa Cruz Biotechnology, Dallas, Texas, USA), TSG101 (1:1000, Abcam, Shanghai, China), ANGPTL1 (1:1000, R&D Systems, Minneapolis, MN, USA). ImKC proteins were detected with the antibodies as follows: STAT3 (1:1000, Cell Signaling Technology, Beverly, MA, USA), phospho-STAT3 (Y705) (1:1000, Cell Signaling Technology, Beverly, MA, USA), JAK2 (1:1000, Cell Signaling Technology, Beverly, MA, USA), phospho-JAK2 (Y1008) (1:1000, Cell Signaling Technology, Beverly, MA, USA), GAPDH (1: 2500, Huabio, Hangzhou, China). HUVECs proteins were detected using the antibodies listed as follows: Claudin-5 (1:1000, Abcam, Shanghai, China), ZO-1 (1:1000, Proteintech Group, Rosemont, USA). All samples were further incubated with the peroxidase-conjugated secondary antibody (1:5000, Huabio, Hangzhou, China). Bands were visualized using enhanced chemiluminescence reagents (YEASEN, Shanghai, China) and scanned via a Tanon 5200 Chemiluminescent Imaging System (Tanon, Shanghai, China). Quantitative analysis of WB was performed with ImageJ software (ImageJ v1.8.0).

In order to detect the protein level in the CM of ImKC through ELISA, cells were cultured in the presence of exosomes (10 μg/mL) for 24 h before supernatants were collected, and the MMP9 level was measured by mouse MMP9 ELISA kit (R&D Systems, Minneapolis, MN, USA).

### Immunofluorescence

For histological analysis, tissues were dissected and fixed in a mix of 2% Paraformaldehyde (PFA) and 20% sucrose solution overnight, followed by embedding and section. Then 6 μm O.C.T. tissue cryosections were stained with antibodies against F4/80 (1:100, eBioscience, Shanghai, China), αSMA (1:500, Abcam, Shanghai, China), CD31 (1:200, Abcam, Shanghai, China), MMP9 (1:200, Abcam, Shanghai, China). Secondary antibodies conjugated to Alexa Fluor 488, 555, or 594 were used (1:500, Abcam, Shanghai, China). Fluorescent images were obtained using a Zeiss LSM 710 laser confocal microscope (Carl Zeiss, Germany) and analyzed using Zen software (ZEN 3.0).

For ZO-1 detection in HUVECs, cell immunofluorescence was conducted as described previously [32]. Cells were incubated with primary antibody ZO-1 (1:400, Proteintech Group, Rosemont, USA) at 4 °C overnight. Nuclei were stained with DAPI for 5 min. Cells were viewed under the Zeiss LSM 710 laser confocal microscope (Carl Zeiss, Germany) and analyzed using Zen software (ZEN 3.0).

### Statistics

All data are presented as the mean ± standard error of the mean (SEM) or mean ± standard deviation of the mean (SD). Statistical analyses were performed with Student’s t-test for comparisons between two groups and with ANOVA for more than two groups using Prism 8.0. *P* < 0.05 was considered statistically significant.

## Results

### ANGPTL1 level was downregulated in CRC derived exosomes

**Fig. 1:**
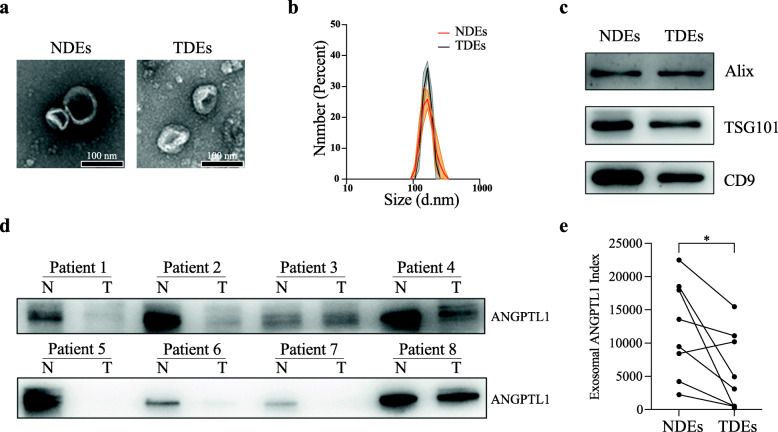
ANGPTL1 level was downregulated in CRC derived exosomes. **a** Transmission electron microscopy analysis of TDEs and NDEs. Scale bar, 100 nm. **b** The number of particles and median diameter was determined by dynamic light scattering (DLS) analysis. Data were shown as mean ± standard deviation of three technical replicates. **c** Western blot (WB) analysis of exosome biomarkers (Alix, TSG101, and CD9). **d, e** WB analysis of the ANGPTL1 in tissue-derived exosomes, *n* = 8, *P* = 0.03. ANGPTL1 level was downregulated in exosomes derived from CRC tumors than paired normal tissues. Data were analyzed using a ratio paired t-test

To study the ANGPTL1 expression in CRC-derived exosomes, we first detected the ANGPTL1 protein level in exosomes secreted by CRC tumors (TDEs) and paired normal tissues (NDEs). We collected eight pairs of samples from our oncology center and isolated the tissue-derived exosomes by ultracentrifugation. The characteristic of TDEs and NDEs were accessed by TEM, DLS, and Western Blot (WB) (Fig. 1a-c). We observed a cup-shaped structure in both TDEs and NDEs, with the diameter of most particles around 150 nm. WB analysis of the exosomal proteins showed that ANGPTL1 level was significantly decreased in TDEs than in paired NDEs (*P* = 0.03, Fig. 1d and e, see the detailed ANGPTL1 index of each sample in additional file 4 Table S1). What is more, the ANGPTL1 level in TDEs varied among CRC patients, indicating the patient heterogeneity of exosomal ANGPTL1 levels. The above results suggested that ANGPTL1 was downregulated in CRC derived exosomes, and exosomal ANGPTL1 may be involved in CRC progression.

### Exosomal ANGPTL1 attenuated liver metastasis and impeded vascular leakiness in the liver PMN

In our previous study [6], ANGPTL1 overexpression inhibited the migration and invasion of CRC cells and hindered liver metastasis. To further investigate the function of exosomal ANGPTL1 in CRC, SW620-ANGPTL1 cells were used to enrich the exosomal ANGPTL1. Exosomes were collected from the CM of SW620 (named Ctrl-Exo) and SW620-ANGPTL1 (named ANGPTL1-Exo) through ultracentrifugation (Additional file 1 Fig. S1a and S1b). WB analysis confirmed that ANGPTL1 was abundant in ANGPTL1-Exo (Additional file 1 Fig. S1c).

**Fig. 2:**
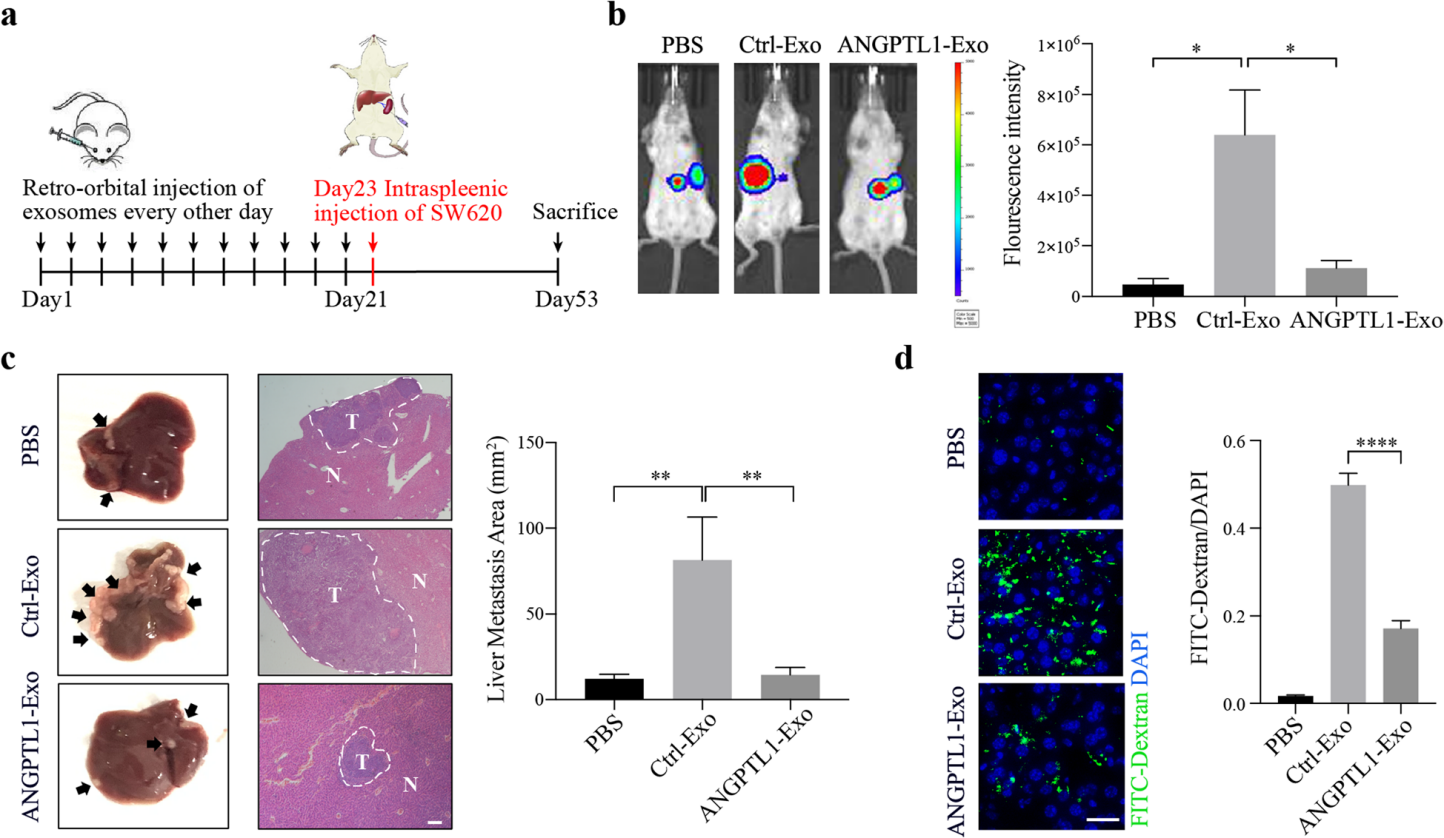
Exosomal ANGPTL1 attenuated liver metastasis and impeded vascular leakiness in the liver PMN. **a** Experimental scheme of exosome education and intrasplenic injection mouse model. **b** Liver metastasis was monitored by the IVIS Lumina imaging system. Represent images (left) and the fluorescence intensity (right; *n* = 3) of the mice livers at the fourth-week post tumor injection are shown. **c** H&E examination (left, arrows indicate tumors) and lesion areas (right; *n* = 5) of mice livers were used for metastatic burden assessment. Scale bar, 200 um. **d** In vivo vascular permeability measured by the appearance FITC-Dextran (green) injected by tail vein (left; n = 3). Data were presented as FITC-dextran /DAPI ratio (right). Scale bar, 20 um. Error bar indicates SEM of the mean. Data in panel b and d were analyzed using t-test, in panel c were analyzed using the Mann-Whitney test. *P < 0.05, **P < 0.01, ****P < 0.0001

We then employed an intrasplenic injection mouse model to investigate the role of exosomal ANGPTL1 in liver metastasis. Firstly, SCID-Beige mice were injected retro-orbitally every other day for 21 days with PBS, Ctrl-Exo, or ANGPTL1-Exo in a process that is defined as “education” (Fig. 2a). After that, 2 × 10^6^ SW620 cells were injected into the mouse spleens. The IVIS Lumina imaging system was used to monitor the liver metastasis weekly. At the fourth week, we found the fluorescence intensity in mouse livers was lower in the ANGPTL1-Exo education group, compared with the Ctrl-Exo education group (*P* = 0.02, Fig. 2b). Then mice were sacrificed, and their livers were harvested for H&E examination (Fig. 2c). The liver macrometastatic burden was measured by lesion areas. We found a heavier metastatic burden in mouse livers educated by Ctrl-Exo than by PBS or ANGPTL1-Exo (PBS vs Ctrl-Exo, *P* < 0.01; Ctrl-Exo vs ANGPTL1-Exo, *P* < 0.01; Fig. 2c). The results suggested that exosomal ANGPTL1 attenuated liver metastasis induced by tumor-derived exosomes in CRC.

It is known that cancer cells spread through the body in multiple steps [33]. Our intrasplenic mouse model allowed tumor cells to immediately disseminate through the portal circulation after injection [30], making tumor extravasation the first step during liver metastasis. Vascular permeability is one of the most critical factors in this process, and a typical characteristic of PMNs [34]. Furthermore, ANGPTL1 was proved to regulate angiogenesis [7]. Thus, we focused on exosomal ANGPTL1’s function on liver vascular permeability. The effect of Ctrl-Exo and ANGPTL1-Exo on endothelial barriers was then detected in vivo. Mice educated by PBS or exosomes were injected with FITC-Dextran through tail veins. More fluorescence was detected in the liver educated by Ctrl-Exo than by PBS. In comparison, less fluorescence was detected in the liver educated by ANGPTL1-Exo (*P* < 0.001, Fig. 2d), which indicated that exosomal ANGPTL1 could impede the vascular leakiness in liver PMNs induced by tumor-derived exosomes.

### Exosomal ANGPTL1 was mainly taken up by KCs and regulated KCs secretion pattern

**Fig. 3:**
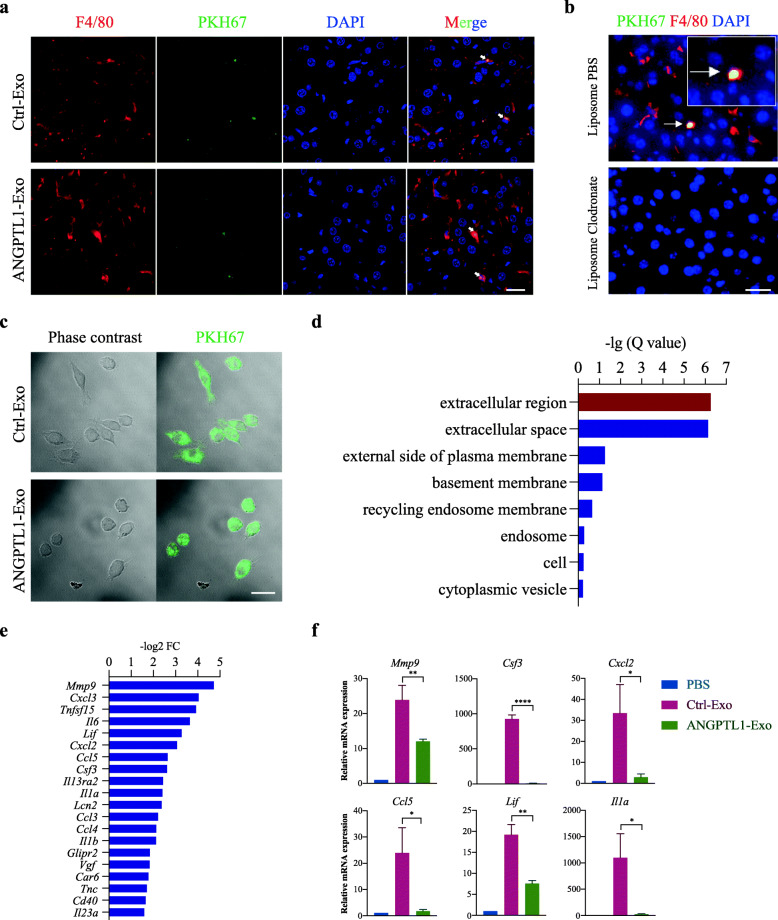
Exosomal ANGPTL1 was mainly taken up by KCs and regulated KCs secretion pattern. **a** Immunofluorescence analysis of PKH67-labeled exosome uptaken. Arrows indicate PKH67-labeled exosomes (green) in F4/80 positive KCs (red). **b** Mice were injected with liposome PBS (up) or liposome clodronate (low) for 2 days. Arrows indicate PKH67-labeled exosomes in F4/80^+^ KCs (yellow). **c** In vitro exosome uptake by ImKC at 4 h was observed on confocal microscopy. Phase contrast (left) and merged (right) pictures are shown. **d** GO enrichment analysis of changing genes between Ctrl-Exo education and ANGPTL1-Exo education group. **e** List of the 20 most significantly downregulated genes is shown (Q < 0.001). **f** qRT-PCR verification of the mRNA expression of *Mmp9, Lif, Cxcl2, Csf3, Il1a, Ccl5*. Scale bar represents 20um. Data are presented as the mean ± SEM of three independent experiments, and analyzed using t-test. *P < 0.05, **P < 0.01, ****P < 0.0001

To explore the mechanism of how exosomal ANGPTL1 inhibits CRC liver metastasis, we first try to determine the cells that take up CRC derived exosomes in the liver. The PKH67 labeled Ctrl-Exo and ANGPTL1-Exo were injected intravenously. Twenty-four hours post-injection, mouse livers were harvested, and liver frozen sections were analyzed by immunofluorescence. We found the cells which took up exosomes were mainly F4/80 positive, a surface marker of KCs (Fig. 3a). Besides, the labeled exosomes failed to fuse with other cells in the liver microenvironment, such as αSMA^+^ hepatic stellate cells or CD31^+^ endothelial cells (Additional file 2 Fig. S2a and S2b). To validate the KC-specific localization of exosomes, we treated the mice with liposome clodronate, known to deplete macrophages, via tail vein injection. We found that 48 h of treatment was enough to ablate most of the F4/80^+^ cells in the mouse liver (Additional file 2 Fig. S2c). Then, the PKH67 labeled exosomes were injected into the mice. We found no exosome remained in livers after macrophage ablation (Fig. 3b), indicating that KC is the predominant cell taking up CRC derived exosomes. Also, we examined the exosome uptake of KCs in vitro. PKH67 labeled Ctrl-Exo and ANGPTL1-Exo were co-cultured with ImKC and were both taken by ImKC in 2 h (Additional file 2 Fig. S2d) and stably fused into ImKC in 4 h (Fig. 3c).

The above results implied that exosomal ANGPTL1 might inhibit CRC liver metastasis through regulating KCs. To further investigate the effect of exosomal ANGPTL1 on KCs, we educated ImKC with PBS, Ctrl-Exo, and ANGPTL1-Exo in vitro for 24 h and analyzed gene expression by mRNA sequencing. GO enrichment analysis showed that the changing genes between ImKC educated by Ctrl-Exo and ANGPTL1-Exo mainly belonged to the extracellular region (Fig. 3d), which suggested that the secretion pattern of KCs was regulated. Among the top20 genes (Fig. 3e), *Mmp9, Lif, Cxcl2, Csf3, Il1a,* and *Ccl5* were verified by qRT-PCR (Fig. 3f, *P* < 0.05). The six genes were significantly downregulated in ImKC educated by ANGPTL1-Exo, and all of them were reported to be involved in PMNs formation. Taken together, we found that exosomal ANGPTL1 was mainly taken up by KCs and regulated KCs secretion pattern, which may result in liver PMNs remodeling.

### Exosomal ANGPTL1 dependent MMP9 decrease in KCs normalized vascular leakiness induced by CRC derived exosomes

**Fig. 4:**
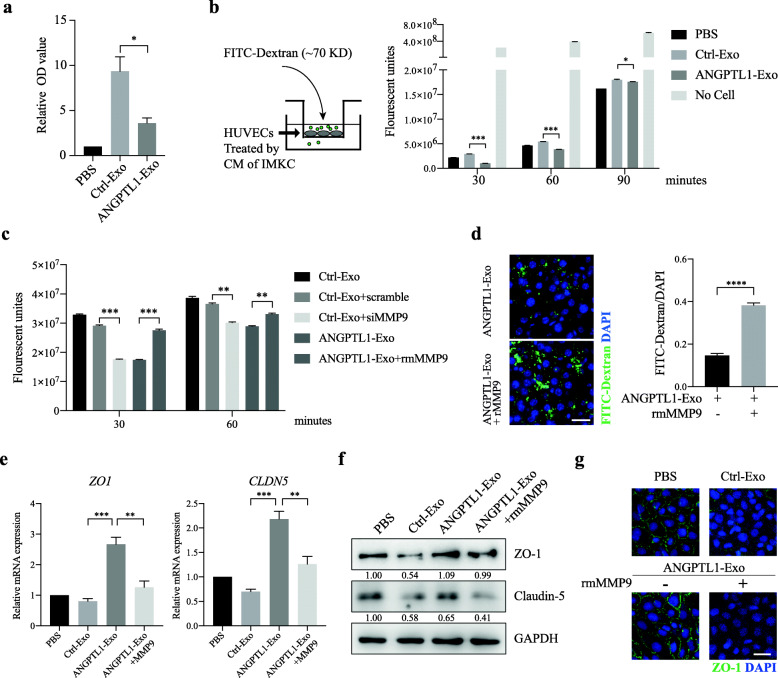
Exosomal ANGPTL1 dependent MMP9 decrease normalized vascular leakiness induced by CRC derived exosomes. **a** ELISA analysis of MMP9 level in the culture medium (CM) from ImKC educated by PBS, Ctrl-Exo or ANGPTL1-Exo for 24 h. **b**, **c** Permeability of the HUVEC monolayers to FITC–Dextran (70 kDa) after exposure to CM from ImKC educated by PBS, Ctrl-Exo, ANGPTL1-Exo (b), or exposure to CM from ImKC educated by Ctrl-Exo followed by transfection of control siRNA (scramble) or MMP9 siRNA or by ANGPTL1-Exo with extra rmMMP9 (100 ng/mL) (c). The fluorescence in the bottom well was detected every 30 min. **d** rmMMP9 (50 μg/kg) was injected through the tail vein into the mice educated by ANGPTL1-Exo for 1 h. In vivo vascular permeability was then determined (n = 3). Representative images are shown (left). Data were presented as FITC-dextran /DAPI ratio (right). **e**, **f** ZO-1 and Claudin-5 expression in HUVECs treated by ImKC CM were detected through qRT-PCR (e) and WB (f). **g** Immunofluorescence staining of ZO-1 in HUVECs monolayers treated by ImKC CM. Data are presented as the mean ± SEM of three independent experiments. Data in panel a, d and e were analyzed using t-test, in panel b and c were analyzed using ANOVA. The scale bar represents 20 um. *P < 0.05, **P < 0.01, ***P < 0.001, ****P < 0.0001

Matrix Metallopeptidase 9 (MMP9) is intimately involved in regulating vascular integrity in PMNs [23]. To explore the effect of exosomal ANGPTL1 dependent MMP9 decrease on vascular leakiness, we first verified the MMP9 downregulation in KCs. The ELISA analysis showed that MMP9 was significantly decreased in the CM of ImKC educated by ANGPTL1-Exo than by Ctrl-Exo (Fig. 4a, *P* < 0.05). We also confirmed that the MMP9 decrease in ANGPTL1-Exo educated mouse livers (Additional file 3 Fig. S3a and S3b). Next, we accessed the permeability of endothelial monolayer after treatment with the CM of ImKC by measuring the passage of FITC-Dextran (70 KD). We found that the CM of ANGPTL1-Exo educated ImKC significantly decreased fluorescent probe leakiness as compared to the CM of Ctrl-Exo educated ImKC (Fig. 4b). We then silenced MMP9 expression in ImKC by siRNA transfection 24 h after Ctrl-Exo education (Additional file 3 Fig. S3c). The CM from MMP9 silencing ImKC caused less fluorescence passage than scramble siRNA (Fig. 4c), which was consistent with the effect of the CM from ANGPTL1-Exo educated ImKC. Moreover, active rmMMP9 was added into the CM of ImKC educated by ANGPTL1-Exo and resulted in more fluorescent probe passing through the endothelial cell (EC) layer (Fig. 4c). The same tendency was observed in the in vivo vascular permeability assay, and rmMMP9 treatment deteriorated liver vascular leakiness in the mice educated by ANGPTL1-Exo (*P* < 0.001, Fig. 4d).

Since the tight junction proteins (TJs) were proved to regulate the EC layer permeability [35], we examined the TJs levels in ECs. The results showed that ZO-1 and Claudin-5 was upregulated in HUVECs treated by the CM of ImKC educated by ANGPTL1-Exo than by Ctrl-Exo. The effect was abolished by extra rmMMP9 (Fig. 4e and f). Immunofluorescence analysis also revealed that rmMMP9 dampened the ZO-1 upregulation in HUVEC monolayers after treatment with CM of ANGPTL1-Exo educated ImKC (Fig. 4g). All the above data above indicated that MMP9 was involved in exosomal ANGPTL1 dependent vascular leakiness prevention.

### Exosomal ANGPTL1 downregulated MMP9 in KCs by inhibiting the JAK2-STAT3 signaling pathway

**Fig. 5:**
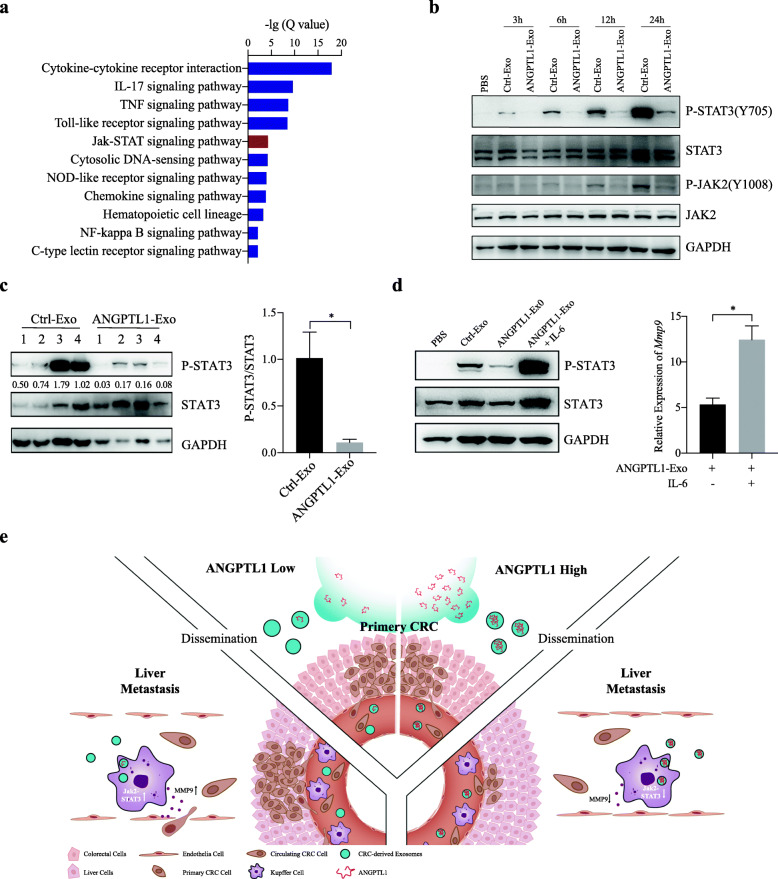
Exosomal ANGPTL1 decrease MMP9 production in KCs by inhibiting the JAK2-STAT3 signaling pathway. **a** KEGG pathway enrichment analysis of the changing genes between ImKC educated by Ctrl-Exo and ANGPTL1-Exo. The top 11 significant pathways were shown. **b** WB validation of the JAK2-STAT3 pathway in IMK educated by PBS, Ctrl-Exo and ANGPTL1-Exo at different times. **c** WB analysis of STAT3 phosphorylation in liver tissues from mice educated by PBS, Ctrl-Exo and ANGPTL1-Exo for 3 weeks (right). The number under the first band indicates the value of P-STAT3/STAT3 and the data were analyzed using t-test (left). **d** WB validation of STAT3 activation induced by IL-6 (left) and qRT-PCR analysis of MMP9 expression (right) in ImKC treated by ANGPTL1-Exo with or without IL-6 for 24 h. **e** Schematic diagram of the role of exosomal ANGPTL1 in CRC liver metastasis. Data are presented as the mean ± SEM of three independent experiments, and analyzed using t-test. *P < 0.05

To further investigate how exosomal ANGPTL1 downregulated MMP9 expression in KCs, we conducted KEGG enrichment to analyze the RNA sequencing data. The results showed that the changing genes between ImKC educated by Ctrl-Exo and ANGPTL1-Exo were mainly enriched in IL-17, TNF, Toll-like receptor, and JAK-STAT signaling pathway (Fig. 5a). Since ANGPTL1 was reported to inhibit the JAK2-STAT3 pathway [18], we focused on the effect of exosomal ANGPTL1 on the JAK2-STAT3 pathway. WB analysis showed that ANGPTL1-Exo obviously inhibited the phosphorylation of STAT3 (Y705) and JAK2 (Y1008) in KCs at 6, 12, 24 h (Fig. 5b and c). MMP9 is known as one of the target genes of STAT3 [36]. To confirm if the JAK2-STAT3 signaling pathway involved in exosomal ANGPTL1 induced MMP9 downregulation in KCs, the recombinant mouse IL-6 was used to active ImKC, which is a specific activator of STAT3. The WB results showed that IL-6 activated the phosphorylation of STAT3 (Y705) and increased the MMP9 mRNA expression in ImKC educated by ANGTPL1-Exo in 24 h (Fig. 5d). The above data demonstrated that exosomal ANGPTL1 downregulated MMP9 in KCs by inhibiting the JAK2-STAT3 pathway, which may be the mechanism of exosomal ANGPTL1 dependent vascular leakiness prevention and liver metastasis attenuation.

## Discussion

ANGPTL1 has been reported to suppress tumor metastasis in several cancers [7], while its extracellular effects on the PMNs are still unclear. Our present study was intended to determine the function of exosomal ANGPTL1 in CRC liver metastasis.

In this study, we found that ANGPTL1 expression was downregulated in the exosomes derived from CRC tumor tissues than paired normal colorectal tissues. The exosomes containing more ANGPTL1 proteins attenuated liver metastasis and impeded vascular leakiness. Further exploration showed that exosomal ANGPTL1 regulated the KCs secretion pattern, significantly decreased the MMP9 expression by inhibiting the JAK2-STAT3 pathway, which in turn normalized vascular leakiness in live PMN (Fig. 5e).

An increasing number of studies have found that ANGPTL1 could be secreted in exosomes. Sinha A et al. identified ANGPTL1 in human ovarian cancer cell-derived exosomes by mass spectrometry [28]. ANGPTL1 was also detected in exosomes derived from human saliva [26] and urine [27]. However, the role of exosomal ANGPTL1 is unknown. Our study demonstrated for the first time that ANGPTL1 expression was downregulated in exosomes derived from CRC tumors than paired normal tissues. It was similar to its differential expression in CRC tumors and normal tissues [6, 37], suggesting a possible suppression role of exosomal ANGPTL1 on CRC progression.

Our functional experiments in mouse models of CRC proved that the exosomal ANGPTL1 upregulation inhibited liver metastasis. Consistently, early studies have proved that ANGPTL1 hindered tumor metastasis in lung cancer [16], hepatocellular carcinoma (HCC) [18], and CRC [6]. But the mechanism varies. Actually, cancer metastasis is a multi-step process [33]. So far, researchers mostly focus on ANGPTL1’s impact on the primary sites, such as tumor invasiveness and mobility inhibition, which prevent tumor cells from invading nearby tissues and moving through the vascular walls [15, 16, 18]. However, we paid our attention to the metastatic organs and found exosomal ANGPTL1 impeded liver vascular leakiness induced by CRC derived exosomes. Growing evidence indicates that exosomes serve as mediators for long-distance cell-to-cell communication and play a pivotal part in PMNs formation [20]. Several studies have shown increased vascular permeability at PMNs, including liver PMN, which is associated with an enhanced metastatic burden [34]. For example, the melanoma-derived exosomes increased the metastatic behavior of primary tumors by inducing vascular leakiness at pre-metastatic sites [38]; breast cancer-derived exosomes destroyed vascular endothelial barriers to promote metastasis [39]. Thus, it may be reasonable to imply that exosomal ANGPTL1 attenuated CRC liver metastasis by preventing liver vascular leakiness. However, whether exosomal ANGPTL1 affected vascular permeability through a direct effect on the endothelial cells or other indirect way is still unknown.

Our study found that KC was the predominant cell that took up CRC-derived exosomes in the liver, which was also approved by Shao Yingkuan et al. [40]. It indicated that exosomal ANGPTL1 possibly remodeled the liver PMNs through KCs. Several studies have correlated KCs with PMNs formation and liver metastasis [41, 42]. Once reprogrammed by tumor-derived exosomes, KCs exert their regurgitation-feeding activity on liver microenvironments via the secretion of cytokines and chemokines [40, 43]. We detected an evident variation of the secretion profile of factors from KCs upon exposure to exosomal ANGPTL1, which influenced the liver PMN formation. Among these changing factors, we attributed MMP9 to exosomal ANGPTL1 dependent vascular leakiness prevention. The ANGPTLs were reported to regulate the expression of MMP9 in osteosarcoma [44] and HCC [45]. Moreover, MMP9 is known to be involved in regulating vascular integrity in PMNs [23]. High levels of MMP9 in the pre-metastatic lung promoted vascular remodeling, while genetic ablation of MMP9 normalized the aberrant vasculature in the lung PMN, impeding cancer metastasis [46]. The above evidence suggested that exosomal ANGPTL1 reprogrammed KC and downregulated its MMP9 expression, thus preventing liver vascular leakiness and hindering CRC liver metastasis.

Like proteins or microRNAs, exosomal cargos can regulate the recipient cells’ physiological activities [47]. We demonstrated that exosomal ANGPTL1 inhibited the JAK2-STAT3 signal pathway, especially hindered STAT3 activation. The exosomal ANGPTL1 dependent MMP9 downregulation was reversed by IL-6 induced STAT3 activation. It indicated that exosomal ANGPTL1 downregulated the MMP9 expression by inhibiting the JAK2-STAT3 signaling pathway. Consistently, Qian Yan et al. also found ANGPTL1 repressed the JAK-STAT3 signaling in HCC [18]. There is substantial evidence confirming that the JAK-STAT3 signaling pathway is involved in CRC development [48]. STAT3 (Signal transducer and activator of transcription 3) is a critical transcriptional factor identified as a central regulator of tumor metastasis. STAT3 could be activated by IL-6 and promote gene transcription, including MMP9 [36]. Therefore, we implied that exosomal ANGPTL1 might influence liver vascular permeability through the JAK2-STAT3-MMP9 axis.

However, how exosomal ANGPTL1 inhibited the JAK2-STAT3 pathway still needs further investigation. The way that exosomes transfer the content or induce signals may involve ligand-receptor interaction or cytomembrane fusion [20]. Several studies have shown that ANGPTL proteins deliver their signals via integrin receptor-related pathways [16, 18]. The previous study proved that ANGPTL1 interacted with the integrin α1β1 receptor to suppress the downstream FAK/Src-JAK-STAT3 signaling pathway [18]. Besides, ANGPTL1 worked intracellular to represses the Src-JAK-STAT3 signaling [18]. Therefore, we implied that the possible mechanism of exosomal ANGPTL1 inhibiting JAK2-STAT3 pathway might be: (1) ANGPTL1 exists on the exosome membrane and binds the integrin receptors on the KCs membrane, suppressing the downstream JAK2-STAT3 signaling pathway; (2) exosomes fuse with the KCs membrane, and ANGPTL1 is released into the cytoplasm, inhibiting the JAK2-STAT3 pathway through interaction with molecules involved in the pathway. These questions will be explored in our future research.

## Conclusion

This study aimed to explore the function of exosomal ANGPTL1 in CRC liver metastasis. Our experimental results demonstrated that ANGPTL1 was downregulated in CRC derived exosomes. More importantly, exosomal ANGPTL1 attenuated CRC liver metastasis and impeded vascular leakiness. In mechanism, we found exosomal reprogrammed the Kupffer cell and decreased MMP9 expression through inhibiting the JAK2-STAT3 signaling pathway. These findings suggest a suppression role of exosomal ANGPTL1 on CRC progression, and expand the approach of ANGPTL1 functioning, enriching the mechanisms of CRC liver metastasis.

## Supplementary Information

**Additional file 1: Fig. S1.** Characterization of exosomes derived from SW620-Ctrl and SW620-ANGPTL1 cells. a Electron microscopy analysis of Ctrl-Exo and ANGPTL1-Exo. Scale bar, 100 nm. b The number of particles and median diameter was determined by dynamic light scattering (DLS) analysis. *n* = 3. Data were shown as mean ± standard deviation of three technical replicates. c Western blot analysis of exosomes positive biomarkers (Alix, TSG101), negative biomarkers (Calnexin), and exosomal ANGPTL1.

**Additional file 2: Fig. S2.** Immunofluorescent validation of the KC-specific localization of exosomes. a, b Immunofluorescence analysis of the colocalization of PKH67-labelled exosome (green) with αSMA^+^ Hepatic stellate cells (hStCs) (A) or CD31^+^ endothelial cells (ECs) (B) (red). Arrows indicate PKH67-labelled exosomes without fusion with hStCs or ECs. c Mice were injected with PBS (up) or liposome clodronate (low) for 2 days. Immunostaining of F4/80^+^ KCs (red) showed complete ablation of KCs in mouse liver after treated with liposome clodronate. d In vitro exosome uptake by ImKC at 2 h was observed on confocal microscopy. Phase contrast (left) and merged (right) pictures are shown.

**Additional file 3: Fig. S3.** qRT-PCR, immunofluorescent, ELISA validation of MMP9 expression. a qRT-PCR validation of MMP9 expression in liver tissue from the mice educated by PBS, Ctrl-Exo or ANGPTL1-Exo, n = 3. b Immunofluorescence analysis of MMP9 in liver slices from the mice education by PBS, Ctrl-Exo or ANGPTL1-Exo, n = 3. c qRT-PCR (left) and ELISA (right) validation of MMP9 silencing in ImKC. Data are presented as the mean ± SD of three independent experiment, and analyzed using t-test. *P < 0.05.

**Additional file 4: Table S1.** The stages of CRC patients and the corresponding exosomal ANGPTL1 index calculated by ImageJ from WB brands. The CRC stage was based on 7th revised edition of the AJCC Colorectal Cancer.

## Data Availability

All data generated or analyzed during this study are included in this published article and its supplementary information files.

## Abbreviations

CRC: colorectal cancer
CLM: colorectal liver metastasis
PMNs: pre-metastatic niches
PBS: Phosphate buffer saline
OD: Optical density
ELISA: Enzyme-linked immunosorbent assay
SD: Standard deviation
SEM: standard error of the mean
ANGPTL1: Angiopoietin-like protein 1
HCC: Hepatocellular Carcinoma
PDAC: Pancreatic ductal adenocarcinoma
KCs: Kupffer cells
HUVEC: Human umbilical vein endothelial cells
ImKC: Immortalized Mouse Kupffer Cells
TDEs: tumor-derived exosomes
NDEs: normal tissue-derived exosomes
TEM: Transmission electron microscopy
DLS: dynamic light scattering
rmMMP9: recombinant mouse MMP9
CM: culture medium
AMPA: aminophenylmercuricacetate
EC: endothelial cell
TJs: tight junction proteins
KEGG: Kyoto Encyclopedia of Genes and Genomes
GO: Gene Ontology
H&E staining: Hematoxylin and eosin staining
