# Supplementary figures and images for: Exosomal ANGPTL1 attenuates colorectal cancer liver metastasis by regulating Kupffer cell secretion pattern and impeding MMP9 induced vascular leakiness
Source: J Exp Clin Cancer Res. 2021 Jan 7;40:21. doi: 10.1186/s13046-020-01816-3 (PMC7792106; doi:10.1186/s13046-020-01816-3)

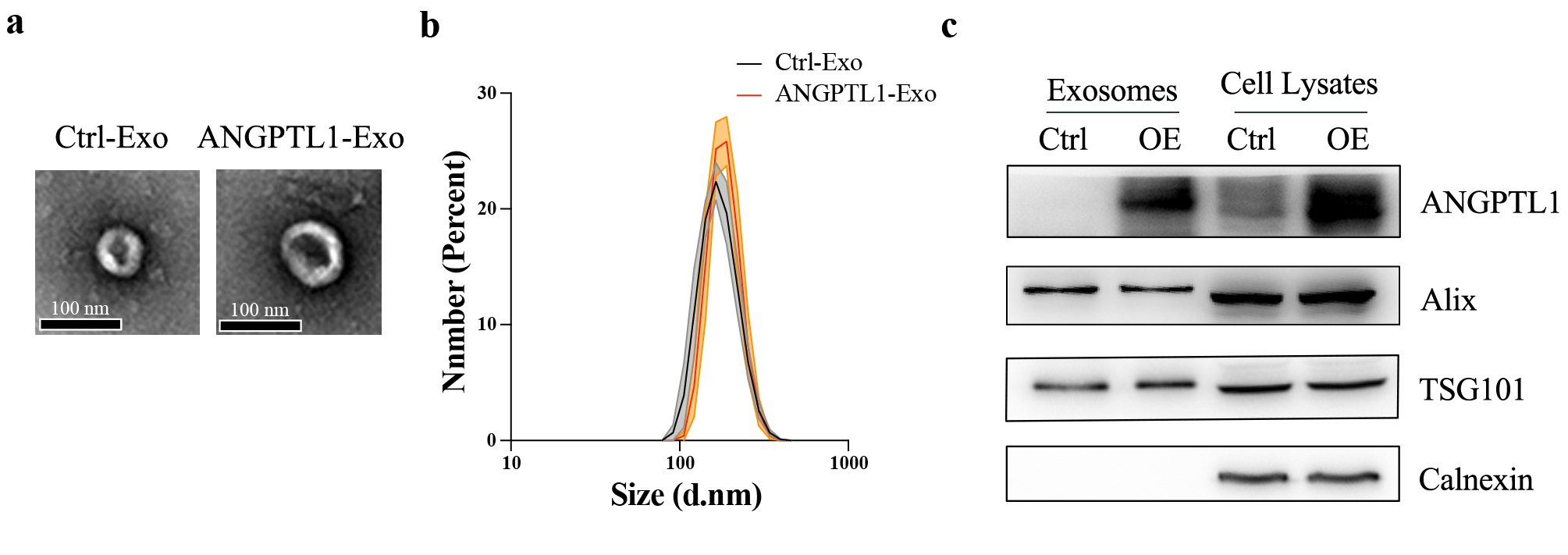

Supplement: Supplementary file 1 — Additional file 1: Fig. S1. Characterization of exosomes derived from SW620-Ctrl and SW620-ANGPTL1 cells. a Electron microscopy analysis of Ctrl-Exo and ANGPTL1-Exo. Scale bar, 100 nm. b The number of particles and median diameter was determined by dynamic light scattering (DLS) analysis. n = 3. Data were shown as mean ± standard deviation of three technical replicates. c Western blot analysis of exosomes positive biomarkers (Alix, TSG101), negative biomarkers (Calnexin), and exosomal ANGPTL1. [file 13046_2020_1816_MOESM1_ESM.tif]

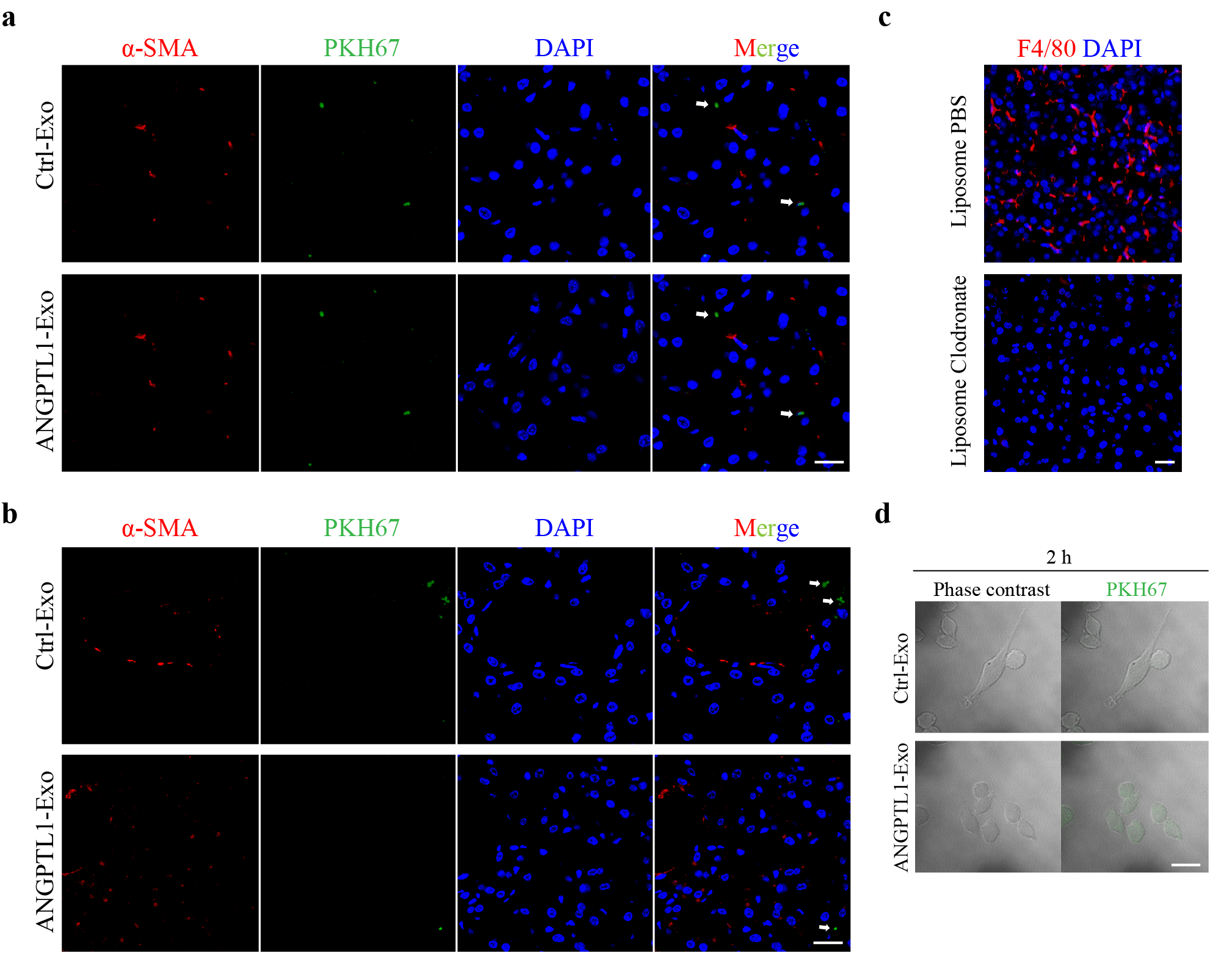

Supplement: Supplementary file 2 — Additional file 2: Fig. S2. Immunofluorescent validation of the KC-specific localization of exosomes. a, b Immunofluorescence analysis of the colocalization of PKH67-labelled exosome (green) with αSMA+ Hepatic stellate cells (hStCs) (A) or CD31+ endothelial cells (ECs) (B) (red). Arrows indicate PKH67-labelled exosomes without fusion with hStCs or ECs. c Mice were injected with PBS (up) or liposome clodronate (low) for 2 days. Immunostaining of F4/80+ KCs (red) showed complete ablation of KCs in mouse liver after treated with liposome clodronate. d In vitro exosome uptake by ImKC at 2 h was observed on confocal microscopy. Phase contrast (left) and merged (right) pictures are shown. [file 13046_2020_1816_MOESM2_ESM.tif]

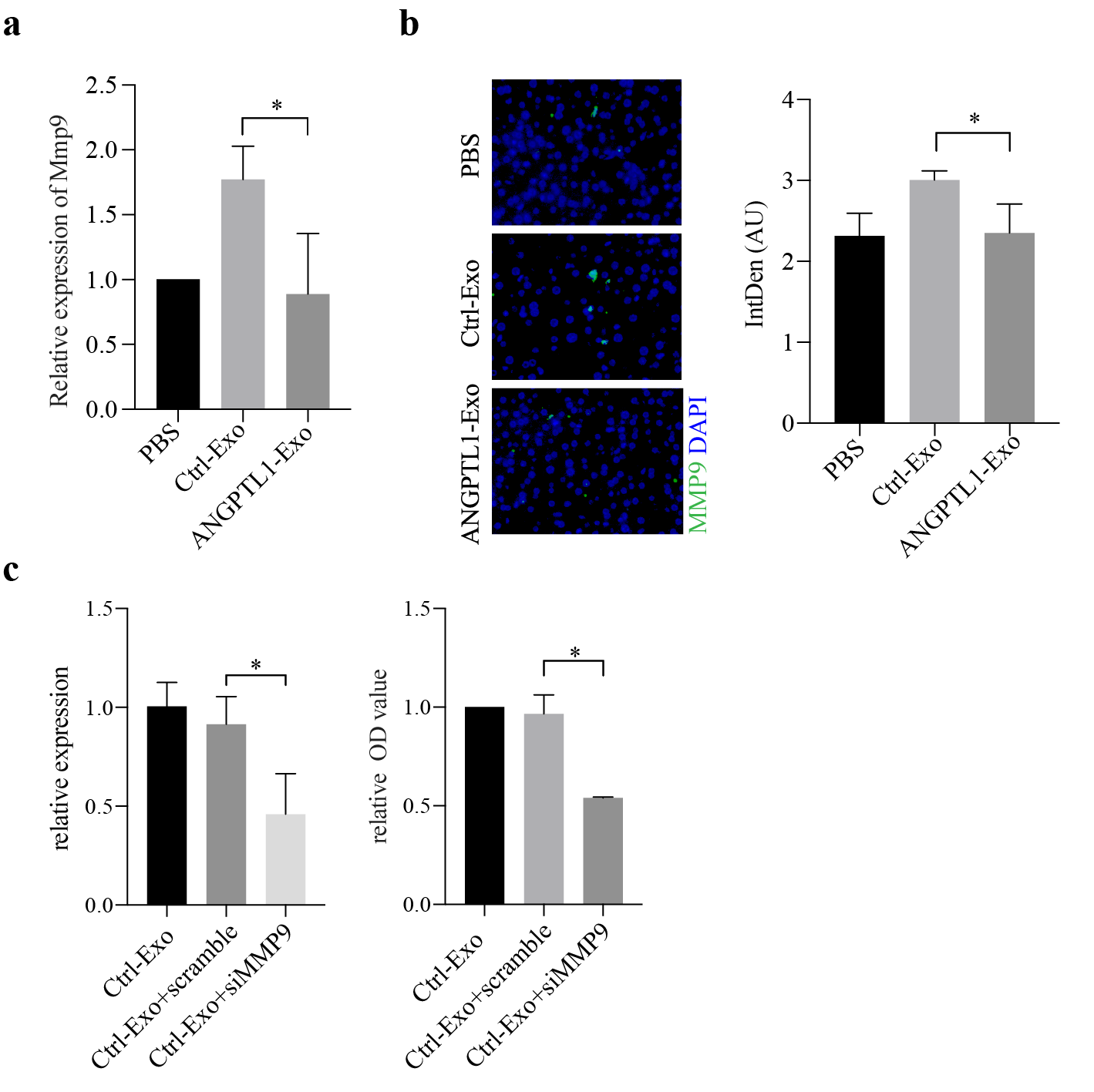

Supplement: Supplementary file 3 — Additional file 3: Fig. S3. qRT-PCR, immunofluorescent, ELISA validation of MMP9 expression. a qRT-PCR validation of MMP9 expression in liver tissue from the mice educated by PBS, Ctrl-Exo or ANGPTL1-Exo, n = 3. b Immunofluorescence analysis of MMP9 in liver slices from the mice education by PBS, Ctrl-Exo or ANGPTL1-Exo, n = 3. c qRT-PCR (left) and ELISA (right) validation of MMP9 silencing in ImKC. Data are presented as the mean ± SD of three independent experiment, and analyzed using t-test. *P < 0.05. [file 13046_2020_1816_MOESM3_ESM.tif]
