# Supplementary material for: Exosomal ANGPTL1 attenuates colorectal cancer liver metastasis by regulating Kupffer cell secretion pattern and impeding MMP9 induced vascular leakiness
Source: J Exp Clin Cancer Res. 2021 Jan 7;40:21. doi: 10.1186/s13046-020-01816-3 (PMC7792106; doi:10.1186/s13046-020-01816-3)
Supplement: Supplementary file 4 — Additional file 4: Table S1. The stages of CRC patients and the corresponding exosomal ANGPTL1 index calculated by ImageJ from WB brands. The CRC stage was based on 7th revised edition of the AJCC Colorectal Cancer. [file 13046_2020_1816_MOESM4_ESM.docx]

Table S1

| Patient | Stage | Exosomal ANGPTL1 Index | |
| --- | --- | --- | --- |
|  |  | **TDEs** | **NDEs** |
| Patient 1 | IIIB | 9459.98 | 3086.13 |
| Patient 2 | IIIA | 18504.59 | 4931.79 |
| Patient 3 | IIA | 8418.93 | 10198.03 |
| Patient 4 | IIIB | 22491.59 | 15467.86 |
| Patient 5 | IIA | 17972.35 | 254.75 |
| Patient 6 | IIIB | 4217.96 | 501.39 |
| Patient 7 | IIA | 2238.43 | 493.21 |
| Patient 8 | I | 13575.47 | 11091.76 |

The stages of CRC patients and the corresponding exosomal ANGPTL1 index calculated by imageJ from WB brands. The CRC stage was based on 7th revised edition of the AJCC Colorectal Cancer.
